# Fully-Automated Volumetric MRI with Normative Ranges: Translation to Clinical Practice

**DOI:** 10.3233/BEN-2009-0226

**Published:** 2009-10-21

**Authors:** J. B. Brewer

**Affiliations:** Department of Radiology and Neurosciences at the University of CaliforniaSan DiegoCAUSA

**Keywords:** Alzheimer’s disease, hippocampus, entorhinal, quantitative, neuroimaging, brain atrophy, temporal horn

## Abstract

Neurodegenerative disorders, such as Alzheimer’s disease (AD), are associated with characteristic patterns of neuropathological spread in the brain. Disease progression is usually accompanied by regional atrophy that can be detected noninvasively using structural magnetic resonance imaging (MRI). A wealth of data has demonstrated the value of quantitative measurements of regional atrophy in AD, suggesting that volumetric MRI (vMRI) may be a useful clinical tool. vMRI provides biological evidence of neurodegenerative disease in patients with cognitive impairment. However, several hurdles impede implementation of vMRI in clinical practice. These include a lack of standardized MRI acquisition protocols, spatial distortions in MRI data, labor-intensive vMRI methods susceptible to interoperator variability, a lack of normative ranges for volume measures, and difficulty integrating vMRI in clinical workflow. Advances in vMRI have resulted from multi-institutional studies of brain imaging, such as the Alzheimer’s Disease Neuroimaging Initiative (ADNI), and help address these challenges. New, fully-automated measures of brain structure volumes coupled with large, multi-center studies using standardized MRI protocols now allow the development of age-adjusted normative ranges for vMRI. Such advances are critical for providing physicians a framework for assessing the pattern and degree of regional atrophy in a patient's brain and applying vMRI in clinical practice.

## 1. Introduction

Noninvasive measurement of disease-related brain atrophy is potentially a powerful tool for early detection and monitoring of neurodegenerative disease, such as Alzheimer's disease (AD). The anatomical detail provided by magnetic resonance imaging (MRI) has long suggested that in vivo examination of patterns of brain atrophy could soon be incorporated into research and clinical practice. Yet more than 25 years after the first commercial MRI scanners were developed, quantitative assessment of subregional brain volumes has yet to be widely used in clinical practice despite a wealth of data demonstrating its promise. Several obstacles have challenged widespread use of these procedures in clinical settings. Labor-intensive methods that require a high degree of expertise and yield operator-dependent results are impractical for the clinical setting. Image formats typically used in research poorly integrate with clinical imaging workflow. Variation in imaging protocols and spatial distortions in MRI data reduce precision of measurements, and normative values have not been available for assessing how an individual's brain structure volumes relate to those of a typical healthy individual. However, researchers have begun to address the above obstacles and recent large, clinical studies have demonstrated the potential for volumetric MRI (vMRI) to be used across sites and scanner vendors. Automated procedures yield operator-independent results in a high-throughput manner, and these procedures may use standard clinical format images as input for easier integration into the clinical imaging workflow. Imaging protocols for volumetry are more widely available and

ISSN 0953-4180/09/$17.00 © 2009 – IOS Press and the authors. All rights reserved correction for spatial distortion can be built into the volumetry procedure. Data from large scale imaging studies can be used to generate normative ranges of brain structure volume, which allow physicians to assess the degree that their patient's volumes differ from those of a matched healthy population. Thus, noninvasive detection of subregional brain structure volume may now be practical for use in the clinical assessment of AD and other neurodegenerative disorders.

The regional progression of neuropathology in patients with AD is well described [1]. Neurofibrillary tangles are seen in the transentorhinal cortex at the earliest stages of the disease and soon spread throughout the anterior parahippocampal gyrus, hippocampus and amygdala. This pathology next spreads to the temporal, parietal and frontal association cortices. Amyloid deposition is first seen in the basal portions of the frontal, temporal and occipital lobes, then spreads more widely to involve nearly all association cortices, with relative sparing of hippocampus and primary sensory and motor cortices. In late stages of the disease, primary sensory and motor regions are involved, as are subcortical structures such as the striatum, thalamus, hypothalamus, subthalamic nucleus, and red nucleus.

The progression of pathology is associated with neuronal dystrophic changes that result in atrophy of affected structures [2] and this atrophy can be detected in vivo using vMRI. Early vMRI studies focused primarily on the hippocampus, a structure that is known to be involved in the early stages of the disease and that is relatively easy to identify and delineate on coronal MRI slices (see Atiya et al. [3] for a review). Approaches to quantify hippocampal atrophy included semi-quantitative visual rating scales [4] and quantitative but labor-intensive manual tracing procedures [5]. Each produced clear evidence that hippocampal volume is associated with subsequent clinical decline in MCI and AD and is associated with a higher rate of conversion from MCI to AD. Indeed, evidence suggests that the inclusion of a biomarker, such as hippocampal volume, in evaluating patients with memory complaints could improve accuracy of diagnosis of early stage AD [6]. However, several challenges must be overcome to allow wider implementation of precise and reliable vMRI.

## 2. Challenges to incorporating vMRI in clinical practice

Intuitively, there is value in providing physicians quantitative data regarding regional structural atrophy in the brains of their cognitively impaired patients, especially in those already undergoing MRI as part of their clinical workup. Despite research supporting the value of vMRI in evaluating cognitive impairment, implementation of this tool in clinical practice faces many hurdles. These include technical challenges to obtaining precise measurement and barriers to acceptance of new clinical data in daily practice, such as the lack of information about sensitivity and specificity in real-world clinical practice and logistical challenges for incorporating the techniques into daily workflow.

### *2.1. Technical hurdles*

#### *2.1.1. Selecting appropriate clinical MRI parameters*

Most clinical radiology practices acquire and interpret two-dimensional MRI data. That is, they acquire images in a particular coronal, sagittal or axial orientation, where in-plane resolution is far greater than slice thickness or through-plane resolution. Indeed, if the focus is solely on qualitative visual inspection of images, two-dimensional acquisition may allow higher patient throughput and more efficient evaluation of images. Three-dimensional or volumetric acquisitions, which allow reformatting of data into any slice plane, is better suited for quantitative analyses, yet these sequences lead to longer scan times and more data to be stored and interpreted. Therefore, they are less commonly used in radiology practice.

Further, segmentation of substructures from MRI images relies partly on delineation of the border between grey and white matter. The MRI parameters selected for acquiring high quality volumetric data must provide consistently high contrast between these tissue types. Achievement of high grey/white contrast has not been a high priority for clinical neuroradiology, where the focus is instead on identifying lesions. In fact, for lesion identification, a bland background that highly contrasts only with lesions might be ideal. Nevertheless, differences in grey/white contrast affects identification of the border between these tissue types, and therefore if grey/white tissue contrast is low, measurement of substructure volumes will be imprecise.

#### *2.1.2. Accounting for spatial distortions in MRI data*

MRI images from most scanners do not accurately represent the spatial dimensions of the object being scanned. Representation of the object's spatial dimensions varies across scanners built by different manufacturers, across scanner versions built by the same manufacturer, and across different equipment and software upgrades for the same scanner [7]. Worse, the spatial distortion may differ based on the positioning of the patient's head relative to the bore of the magnet. Thus, even when scanning the same subject twice using the same equipment and parameters, measurements will likely differ for the two acquisitions. Again, the clinical practice of radiology has not been greatly affected by such spatial distortions, because clinical judgments have not depended upon a fine degree spatial accuracy or consistency in spatial representation across scan sessions. However, quantitative neuroimaging critically depends upon imaging data representing the true spatial dimensions of the brain. Until recently, this was a feature of MRI that was largely overlooked even in vMRI studies.

#### *2.1.3. Quality checks for excessive patient motion and other artifacts*

The population discussed here, elderly patients with cognitive impairment, will have a relatively high percentage of scans with poor image quality due to subject motion. Even if the scan is free of motion artifact, the degree of vascular disease in these patients can be quite extensive. Each of these factors may interfere with image segmentation and therefore, quality checks on the results of segmentation will always be necessary at some level. Development of robust prospective motion correction procedures for use during MRI acquisition will likely assist wider use of vMRI in severely impaired patients by reducing motion artifact.

#### *2.1.4. Increasing throughput and reducing reliance on high-level expertise*

One of the most challenging hurdles has been to increase the throughput of vMRI to make it practical for clinical use. Quantitative vMRI has largely relied upon manual or semiautomated techniques for image segmentation. Such procedures are not practical for clinical use because they are relatively slow, labor intensive, and require a high-degree of expertise. They may also suffer from inter- and intra- operator variability that limits the generalizability across sites. High throughput vMRI procedures that are immune to inter-operator variability will be required to compare results across clinical settings and to gain the high volume of normative data that will be critical to allow clinical interpretation. Thus the procedures should be essentially independent of manual input.

#### *2.1.5. Integrating with clinical workflow*

For acceptance into clinical practice, a vMRI procedure must integrate well with the existing clinical workflow. This is greatly facilitated if the processes maintain compatibility with the image format and data transfer procedures that are universally used in clinical practice, namely the Digital Imaging and Communications in Medicine (DICOM) standard. Ideally, the procedure should be able to accept DICOM format images as input and the final output should also be DICOM compatible, allowing integration with the clinical practice's Picture Archiving and Communication System (PACS). This would allow rapid visualization of segmentation results for quality control and for reporting of the results by clinical personnel.

### *2.2. Additional scientific hurdles*

#### *2.2.1. Gathering normative values*

In order for a physician to interpret biomarker values in patient care, he or she must have a sense of the normal range for the biomarker in a comparable group of healthy patients. Normative values for structures other than the hippocampus are not widely available. Studies on hippocampal volume in patients and age-matched controls exist in the literature, and these studies include quantitative measures that have largely been consistent despite varied techniques and expertise at manual tracing. However, variations in scan procedures and analysis techniques lead to variability that reduces the generalizability of normative ranges. Further, it is expected that sex and intracranial volume should be accounted for in these measures, as volumes may be influenced by these factors [8]. Finally, healthy aging leads to progressive atrophy of the brain, and in particular, the hippocampus. In fact, most studies have noted that the hippocampus atrophies at a rate of approximately 1% per year in healthy aging (compared to around 5% per year in AD [9]), and therefore, vMRI, if performed on a patient-by-patient basis, must use a normative range for controls that are age-matched to the patient. Ideally, normative values would be obtained from a large set of healthy subjects who were each scanned using the same sequence parameters and where vMRI procedures were consistent. The norms should account for variability between scanner manufacturers and additional variability due to hardware specifications and software upgrades.

#### 2.2.2. *Evaluating use of vMRI in the clinical setting*

Research that evaluates the use of vMRI in the clinical setting may be needed before wider clinical use is encouraged. More studies are needed on the use of vMRI in the clinical setting. Most laboratory-based research studies do not adequately represent the diversity of patients seen in the clinic, so clinic-based studies will be valuable to examine the impact of vMRI on patient care in a real-world setting. Such clinic-based research will guide use of the technology and help avoid inappropriate uses of the data, such as relying too heavily on the results of volumetry without appropriate consideration of additional clinical data.

For instance, it remains unclear whether measures of hippocampal atrophy have adequate specificity to distinguish between causes of memory complaints in the elderly. While hippocampal atrophy is profound in AD, and AD is possibly the most common cause of hippocampal atrophy, AD is not the only disorder associated with hippocampal atrophy. Disorders such as schizophrenia [10], traumatic brain injury [11], frontotemporal dementia [12], epilepsy [13] and even depression [14] have been associated with some degree of hippocampal volume loss. Sensitivity and specificity values for hippocampal vMRI in AD are commonly reported in the literature relative to healthy elderly patients, but in clinical practice, the comparison group is rather different and consists of patients with other causes of memory impairment. No study has used vMRI to look across a diverse group of patients, such as that seen in a behavioral neurology clinic, to compare relative degree of hippocampal tissue loss. Therefore, differential diagnosis of patients with cognitive impairment and hippocampal atrophy is likely to remain a challenge that will require correlation with other clinical features. More research is needed to understand the proper weighting that a physician should assign to new clinical information provided by vMRI. It is doubtful, for example, that normal hippocampal volumes could be used in the future to ‘rule out’ AD, especially given variants of the disease that only minimally affect the MTL and damage cortical areas in an atypical pattern [15]. However, normal hippocampal volumes may be reassuring to a high-functioning patient extremely concerned about mild, but increasing memory failures. Whether such reassurance is appropriate or not remains an open question.

## 3. Toward translation of vMRI to the clinical setting

### 3.1. *The Alzheimer’s disease neuroimaging initiative*

The Alzheimer’s Disease Neuroimaging Initiative (ADNI), is a multi-institutional, longitudinal neuroimaging biomarker study of 800 elderly patients comprising 200 elderly controls, 400 patients with prodromal AD or amnestic mild cognitive impairment (MCI), and 200 patients with early stage AD [16]. The planning stages of ADNI gathered some of the foremost experts in quantitative neuroimaging of AD to discuss ways of overcoming the technological hurdles of performing precise vMRI across multiple sites and scanner models [7].

Many of the challenges faced by ADNI are the same as those that must be overcome for clinical translation of vMRI. The selection of scanning parameters that would allow comparable volumetric imaging across multiple platforms and dealing with variability related to spatial distortions across scanner models and software versions were two important challenges that ADNI overcame. ADNI has greatly advanced the development of standardized and optimized scanning procedures for vMRI. In addition, the ADNI database is fully available to the public and can be used to aid the development of normative values for vMRI. The knowledge gained through this important and unprecedented study will fuel further translational vMRI research and speed testing and development of new technologies aimed at bringing vMRI to the clinical setting.

### 3.2. *Piloting clinical vMRI: Experience at UCSD*

At our institution, we have made vMRI available on request as a neuroimaging procedure offered through the department of radiology. This endeavor required coordination between the radiology and neurology departments to assure that any added steps would fit the clinical workflow of both departments. The primary motivation was to serve neurologists who had expressed interest in quantitative assessment of asymmetric hippocampal atrophy associated with epilepsy and bilateral hippocampal atrophy associated with AD. The availability of a current procedural terminology code (CPT 76377, http://www.cms.hhs.gov) for reporting of quantitative segmental volumes was helpful in compensating physicians in the radiology department for the additional work required when the procedure was requested by the referring physician.

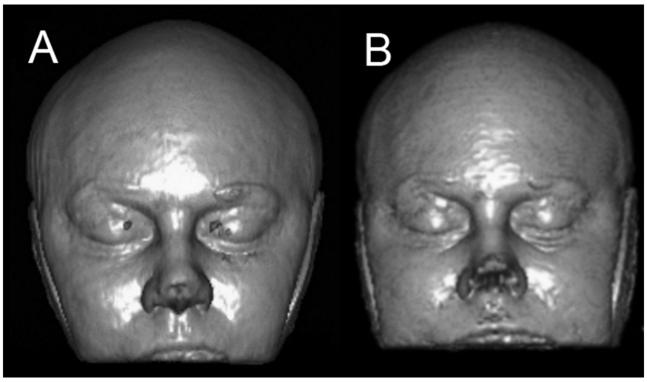

Fig. 1. Three dimensional reconstruction of MRI data A) before correction for spatial distortion and B) after correction. The volume reconstruction after correction is a more accurate representation of the subject's head shape.

Because the Centers for Medicare and Medicaid Services did not have a national coverage policy that addressed the specific use of this procedural code for assessment of dementia, we turned to our regional Medicare office for guidance. We learned that this code is often used inappropriately and that for each use we should carefully document the reason for quantitative segmental volume assessment. Though it is appropriate to bill for this procedure to follow up positive findings noted in MRI, our radiology department uses the procedure conservatively and only performs quantitative segmental volume assessment in dementia when specifically requested by the referring physician.

The software we selected is NeuroQuant [17,18], which provides fully automated segmentations that have been validated against manual methods and which has obtained 510K approval by the Food and Drug Administration as a device for providing quantitative segmental volumes. The procedure makes use of three dimensional T1-weighted MRI datasets with high grey-white contrast to register a patient's brain anatomy to a probabilistic atlas for anatomical labeling. This atlas is similar to that of semi-automated methods commonly used in the research setting [19] but has been designed to better represent the aged population.

#### 3.2.1. *Clinical vMRI parameters*

The MRI protocol used at UCSD for clinical vMRI is similar to that used by ADNI. MRI protocols typically vary by device manufacturer. ADNI made use of a magnetization prepared rapid gradient echo (MPRAGE) sequence on all scanners, but this sequence is not available on clinical scanners manufactured by General Electric. Instead, General Electric provides an inversion recovery spoiled gradient echo (IR-SPGR) sequence that delivers similar grey-white contrast and, in our experience, similar segmentation volumes. The devices used for clinical volumetry at our institution are General Electric 1.5 Tesla scanners. One has Horizon software version 9.1 and another has software version HDx. Our IR-SPGR sequence uses a flip angle of 10 and an inversion time of 500. The images are acquired in approximately 7 minutes.

#### 3.2.2. *Accounting for spatial distortions and image intensity variations*

After acquisition, the three-dimensional T1 volume is sent via the hospital PACS to the NeuroQuant device. The NeuroQuant device includes preprocessing steps that determine that the MRI sequence conforms to specifications required to perform automated segmentation and then performs corrections for scanner specific spatial distortions and image intensity variations caused by gradient nonlinearity and B1 field inhomogeneity. An example of an MRI dataset reconstructed into three dimensions with and without spatial distortion correction is provided in Fig. 1.

#### 3.2.3. *Relating to normative values*

An example of a clinical vMRI report from UCSD is provided in Fig. 2. Briefly, the report provides patient and referral information at the top. A sagittal, coronal, and axial image with color-labeled structures are provided to give the referring physician a sense of the quality of the segmentation and the atrophy seen in the MTL. A table is divided by rows for each structure (Lateral Ventricle, Hippocampus, Temporal Horn) and by columns containing 1) the structure's raw volume in ccs, 2) volume expressed as a percentage of intracranial volume and normative range for the age group, 3) percentile rank for the patient's measurements relative to the normative dataset of healthy subjects aged 50 and

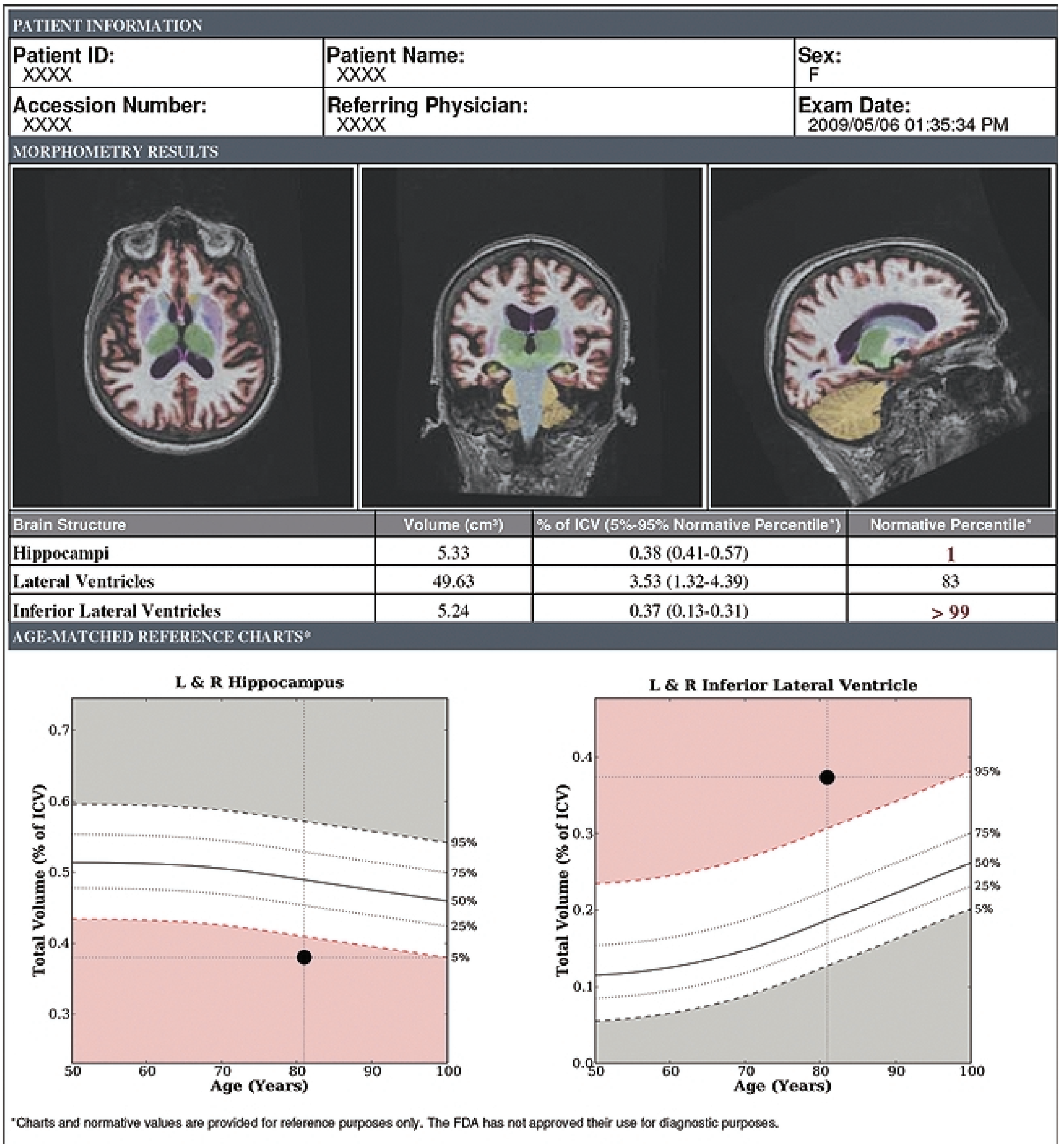

PATIENT INFORMATION

| Patient ID: XXXX | Patient Name: XXXX | Sex: F |
|---|---|---|
| Accession Number: XXXX | Referring Physician: XXXX | Exam Date: 2009/05/06 01:35:34 PM |

MORPHOMETRY RESULTS

| Brain Structure | Volume (cm³) | % of ICV (5%-95% Normative Percentile*) | Normative Percentile* |
|---|---|---|---|
| Hippocampi | 5.33 | 0.38 (0.41-0.57) | 1 |
| Lateral Ventricles | 49.63 | 3.53 (1.32-4.39) | 83 |
| Inferior Lateral Ventricles | 5.24 | 0.37 (0.13-0.31) | > 99 |

AGE-MATCHED REFERENCE CHARTS*

*Charts and normative values are provided for reference purposes only. The FDA has not approved their use for diagnostic purposes.

Fig. 2. Example of a clinical vMRI report. Patient data and the radiologist's interpretation have been removed.

above. Below this are two graphs showing the patient's volumes plotted relative to the age-adjusted normative ranges for the hippocampus and temporal horn. At the bottom, the radiologist or neurologist enters his or her assessment in a text box.

*3.2.4. Integration into clinical workflow*

An often overlooked challenge for incorporating a new technology from the research laboratory into the clinical setting is the integration of the procedure into the clinical workflow. Figure 3 shows the flow of information for vMRI at UCSD. A referring physician orders the procedure by fax or by electronic order on the electronic medical record system. The request is routed to the MRI center for scheduling on a scanner that has the volumetric protocol. This protocol is a standard clinical sequence, but the imaging parameters have been set for optimal contrast between grey and white

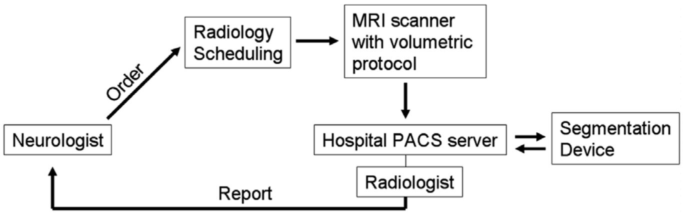

Fig. 3. Example of data flow for clinical vMRI. The referring physician (e.g. neurologist) initiates a request for vMRI. The radiology scheduling center schedules the exam on a scanner with the vMRI protocol for achieving high grey/white contrast. The images are sent to the clinical PACS system where they can be routed to the image segmentation device. The image segmentation device performs image preprocessing including spatial distortion correction, segments the image and generates an initial report relating the patient's volumes to a normative database. The resulting data is routed back to the clinical PACS system, where the radiologist reviews the information, performs quality checks, and generates the final report for the referring physician.

matter, similar to that provided by the ADNI protocol. When a scan is done, the images are sent via PACS to the NeuroQuant device. The software processes the dataset in around 8 minutes, and a new full-volume spatially corrected and anatomically labeled dataset is returned to the PACS along with a volumetric report similar to that of Fig. 2. The images and values are inspected and quality checked. Images are selected for the final report, and the text box is filled in by the interpreting physician via a secure web-interface with the NeuroQuant device. The final report is saved and sent to PACS or printed out as a portable document format (PDF) file for mailing to the referring physician or for upload onto the electronic medical record.

*3.2.5. Initial experience*

Over 6 months, 45 clinical vMRI studies have been requested by referring physicians, all of whom are subspecialists focusing on Alzheimer's disease. Approximately 15 percent of requests have come from outside our typical referral network. Of the 45 patients scanned, two patients had data that was not able to be segmented due to excessive motion artifact. All others passed quality checks and separate vMRI reports, in addition to the primary clinical interpretation of the MRI images, were generated for the referring physicians. Medicare reimbursement for the added procedure has been consistent as long as the primary clinical radiology interpretation makes note of the existence of a separate volumetric report. MediCal does not reimburse for the procedure.

The number of referrals for vMRI has steadily increased and informal feedback from referring physicians has been positive. None of the physicians has withdrawn from referring patients for vMRI. The availability of clinical vMRI at UCSD has widened the referral base of our radiology services to include practices seeking vMRI data on their patients. The informal feedback included discussion that vMRI provides information that is not otherwise available and that is complementary to the history, neurological exam, neuropsychological testing, biofluid tests, and nuclear medicine imaging currently available for evaluating cognitive impairment. In addition, the report provides a visual aid for educating patients and their families. In no case is a physician reporting that they solely rely on vMRI for their diagnosis, but several have found it to be highly consistent with their clinical impressions and helpful in providing a biological foundation for those impressions. Our plan is to conduct a more formal survey on the impact of vMRI on referring physicians' clinical decision making.

## 4. Conclusions

Developments allowing consistency in acquisition of MRI data across sites and fully-automated image segmentation bring closer the promise of clinical vMRI. This promise has long been suggested by single site research studies using manual or semi-automated procedures for volumetry, but a number of hurdles need to be crossed before widespread clinical implementation. Large, multi-site clinical trials, such as ADNI, have helped advance vMRI toward greater use in the clinical setting by providing standardization of image acquisition, correction of spatial distortions in MRI data, improved data throughput, and the possibility of generating large normative databases for brain structure volumes. Fully-automated procedures have reduced reliance on high-level anatomical expertise and avoid interoperator variability while providing rapid turnaround compatible with clinical practice. Such advances, available only recently, will greatly facilitate wider implementation of vMRI beyond the academic setting.
